# Large Language Model–Generated Synthetic Data for Parole and Probation Detection in Clinical Notes: Development and Evaluation Study

**DOI:** 10.2196/104586

**Published:** 2026-10-19

**Authors:** Avijit Mitra, Emily Druhl, Raelene Goodwin, Hong Yu, Matthew A Stimmel, Jack Tsai

**Affiliations:** 1 University of Massachusetts Amherst Amherst, MA United States; 2 United States Department of Veterans Affairs Bedford, MA United States; 3 University of Massachusetts Lowell Lowell, MA United States; 4 United States Department of Veterans Affairs Washington, DC United States; 5 Yale University New Haven, CT United States; 6 The University of Texas at San Antonio Health Science Center San Antonio, TX United States

**Keywords:** parole and probation detection, clinical natural language processing, large language models, synthetic data, electronic health records, legal status classification

## Abstract

**Background:**

Identifying individuals on parole or probation can help inform care pathways and ensure continuity of care in health care systems. However, this sensitive justice-related information is often absent from structured electronic health record fields and is instead documented in narrative clinical notes. Developing automated parole and probation detection (PPD) systems typically requires expert-annotated clinical data, but manual annotation is costly, time-consuming, and constrained by data sparsity and privacy concerns.

**Objective:**

This study aimed to develop and evaluate a synthetic-only training paradigm for PPD from clinical text that eliminates the need for expert-annotated training data while maintaining strong performance and cross-domain transfer to real-world data.

**Methods:**

We developed Synth-PPD, a synthetic dataset comprising 7579 labeled clinical notes generated using large language models (LLMs), redundancy filtering, and automated quality evaluation. Dataset quality was assessed through human review of 1517 synthetic notes by 2 annotators. The primary outcome was the macro–*F*_1_-score for PPD. We evaluated Synth-PPD across unsupervised and supervised learning settings using LLMs, traditional machine learning models, and transformer encoders. Performance was assessed on 293 expert-annotated real-world clinical notes from the US Department of Veterans Affairs.

**Results:**

Synth-PPD achieved 90.05% human-LLM alignment. In the unsupervised setting, Llama 3.3-70B-Instruct with chain-of-thought prompting and few-shot examples from Synth-PPD achieved a macro–*F*_1_-score of 87.22%, outperforming both the corresponding zero-shot model (80.10%) and the best rule-based baseline (77.55%; *P*<.001 for all comparisons). In the supervised setting, Llama 3.1-8B fine-tuned on Synth-PPD achieved a macro–*F*_1_-score of 81.43%, exceeding the best rule-based baseline (77.55%; *P*<.001) and achieving a numerically higher score than the substantially larger zero-shot Llama 3.3-70B-Instruct model (80.10%; *P*=.08). In contrast, traditional machine learning models and transformer encoders exhibited greater performance degradation under domain shift and derived limited benefit from increasing synthetic training data. Additional experiments showed that increasing synthetic training data improved cross-domain performance for LLM-based models but yielded comparatively smaller gains for traditional approaches.

**Conclusions:**

Synthetic data generated by LLMs can serve as a scalable, cost-effective, and privacy-preserving alternative to expert-annotated real-world clinical data for PPD from clinical text. The findings demonstrate successful cross-domain transfer to real-world US Department of Veterans Affairs clinical notes.

## Introduction

Substantial prior research has documented important relationships between criminal justice involvement and health outcomes. Individuals who become involved in the criminal justice system often experience disruptions in health care access due to legal obligations, periods of incarceration, or competing social and administrative demands. Despite its clinical and public health relevance, criminal justice involvement is rarely captured in structured fields within electronic health records even though it is commonly addressed during intake and biopsychosocial assessments in clinical care [1]. As a result, identifying patients affected by parole or probation remains challenging for health care systems seeking to understand and address the health needs of this population. Moreover, limited research has focused on this high-risk population group, and few efforts have sought to identify patients within this group to support service planning and continuity of care. In this study, we addressed this gap by developing a system to identify patients on parole or probation using veterans receiving care in the US Department of Veterans Affairs (VA) health care system—the largest integrated health care system in the country—as a case example.

Military veterans represent a critical subpopulation in the United States from both public health and public safety perspectives. According to the most recent data from the Survey of Prison Inmates conducted by the US Bureau of Justice Statistics, approximately 107,400 veterans were serving time in state or federal prison in 2016 [2]. To address criminal justice involvement and help connect veterans with the care they need, the VA has established 2 major initiatives. The Veterans Justice Outreach program focuses on early intervention to prevent unnecessary incarceration by linking veterans with health care and social services [3]. The Health Care for Re-entry Veterans program, in turn, supports veterans transitioning from incarceration to the community by ensuring access to VA and community services [4]. A growing body of research has examined the needs and outcomes of veterans in both the Veterans Justice Outreach and Health Care for Re-entry Veterans programs [5-7].

Veterans may be involved in the criminal justice system at multiple stages, yet a notable knowledge gap remains regarding those on parole or probation [8,9]. Parole refers to the conditional early release of an incarcerated individual under supervision contingent on compliance with specific conditions. Probation denotes a court-ordered period of community supervision that allows offenders to avoid incarceration if they adhere to defined rules. Thus, parole and probation are specific justice supervision statuses rather than broad indicators of legal problems or justice system involvement. Both statuses serve as critical indicators of psychosocial and health outcomes. Prior studies have found that individuals on parole or probation experience higher disease burden, reduced access to outpatient care, and elevated mental health and substance use treatment needs compared to the general population [10,11]. However, these issues have not been well studied among veterans or within the VA health care system, which provides comprehensive health care to eligible veterans.

This research gap stems primarily from the absence of systematically collected data on parole and probation status in structured electronic health record data. The VA’s structured data systems lack dedicated fields for capturing criminal justice involvement, thereby limiting the ability to identify such cases through conventional means. In contrast, unstructured clinical notes often contain valuable information about patients’ legal circumstances, presenting an alternative resource to extract parole and probation status using natural language processing (NLP). Although broader social and behavioral determinants of health (SBDH) extraction may capture general information about legal or justice system involvement, parole and probation detection (PPD) requires finer-grained identification of specific parole and probation statuses, including distinctions such as negated and affirmative mentions and relevant temporal context. Developing such systems, however, requires a large volume of annotated data, and the sensitive nature of clinical text restricts data sharing. Additionally, manual chart review is labor intensive, costly, and time-consuming, creating substantial barriers to building high-performing, generalizable clinical NLP systems.

Synthetic clinical data offer a promising avenue for addressing challenges related to data scarcity and privacy. Recent advances in large language models (LLMs) have enabled the generation of high-quality synthetic text for a range of health care applications, including SBDH extraction [12-14], patient-physician conversation-to-note generation [15], and medical text summarization [16]. Despite these developments, the potential of synthetic data for detecting criminal justice supervision—specifically parole and probation status—has not been systematically investigated.

The aim of this study was to determine whether models trained exclusively on synthetic clinical text can generalize to real-world clinical documentation. We introduced a framework for PPD that relies entirely on LLM-generated synthetic data for system development, eliminating the need for expert annotation or access to real-world training data. Using this framework, we constructed Synth-PPD, a synthetic clinical dataset designed to capture diverse mentions of parole and probation status, including contextual attributes such as negation and temporal information. We then evaluated multiple LLM-based approaches trained on Synth-PPD and assessed their ability to detect parole and probation status in real clinical notes from the VA health care system.

This work makes 3 primary contributions.

First, we introduce the novel task of PPD from clinical notes. To our knowledge, this is the first study to explore automated identification of these indicators of criminal justice involvement within clinical text.

Second, we developed and released Synth-PPD, the first synthetic clinical dataset for PPD. Synth-PPD was constructed using a scalable pipeline combining LLM generation, redundancy filtering, and LLM-as-a-judge validation, producing diverse annotated examples. Expert evaluation demonstrated strong human-LLM alignment (90.05%), and the expert-annotated test split provides a valuable benchmark for future research on automated PPD systems.

Third, we propose an intuitive and effective PPD framework that relies exclusively on Synth-PPD without human supervision and conducted comprehensive in-domain and out-of-domain evaluations using rule-based systems, traditional machine learning (ML) models, transformer encoders, and LLMs. In both unsupervised and supervised settings, LLMs with Synth-PPD substantially outperformed all other baselines on expert-annotated clinical notes from the VA health care system.

Together, these results highlight the potential of synthetic clinical data as a scalable, privacy-preserving alternative for developing NLP systems in sensitive health care domains where real-world annotated data are scarce.

## Methods

### Synthetic Dataset (Synth-PPD) Construction

Motivated by the data privacy constraints and limited availability of annotated clinical notes containing parole or probation mentions, we constructed a fully synthetic dataset—Synth-PPD. The dataset was generated using a 3-stage automated pipeline without human supervision.

First, we used an LLM (GPT-4o-mini; OpenAI [17]) with few-shot in-context learning (ICL) [18] to generate 10,014 synthetic clinical note excerpts. Each instance was annotated with 4 attributes: *label*, *presence*, *period*, and *rationale*. The *label* attribute indicates whether the mention refers to parole or probation or is unclear. The *presence* field (values: *yes*, *no*, and *unclear*) captures the presence or absence (negation) of the *label* attribute, whereas the *period* field (values: *current*, *history*, *future*, and *unclear*) encodes the temporal context of the mention. *Rationale* states why or how the LLM chose specific *label*, *presence*, and *period* values for an instance.

Second, we reduced redundancy using Recall-Oriented Understudy for Gisting Evaluation (ROUGE)–based similarity filtering [19]. Pairwise similarity was computed using ROUGE-L (lowest common subsequence–based statistics), and examples exceeding a similarity threshold of 0.7 were removed. This filtering step reduced the dataset from 10,014 to 8266 unique examples. Third, we adopted an LLM-as-a-judge framework [20] to automatically assess annotation quality. A second LLM, GPT-4.1 (OpenAI) [21], independently annotated the 8266 examples from the second stage after ROUGE-based filtering. Each example was then assigned a score on a 4-point Likert scale based on agreement between the 2 annotation sets: (1) rating 1: presence does not match; (2) rating 2: presence matches, but label and period do not; (3) rating 3: label and presence match, but period does not; and (4) rating 4: label, presence, and period all match.

After qualitative review of representative examples from each rating category, domain experts considered ratings 3 and 4 to be sufficiently appropriate for inclusion, whereas ratings 1 and 2 did not meet the desired quality threshold—yielding 7579 high-quality instances. For rating 3 examples, the *period* annotations from GPT-4.1 replaced the original annotation. The final dataset was split into training (70%), validation (10%), and test (20%) sets, corresponding to 5305, 757, and 1517 examples, respectively. The test set was subsequently reviewed and reannotated (if necessary) by 2 human experts for quality assessment. Human review was restricted to the held-out test set to provide an independent quality assessment without requiring exhaustive expert annotation of the full Synth-PPD corpus. The Synth-PPD generation pipeline is illustrated in Figure 1.

**Figure 1:**
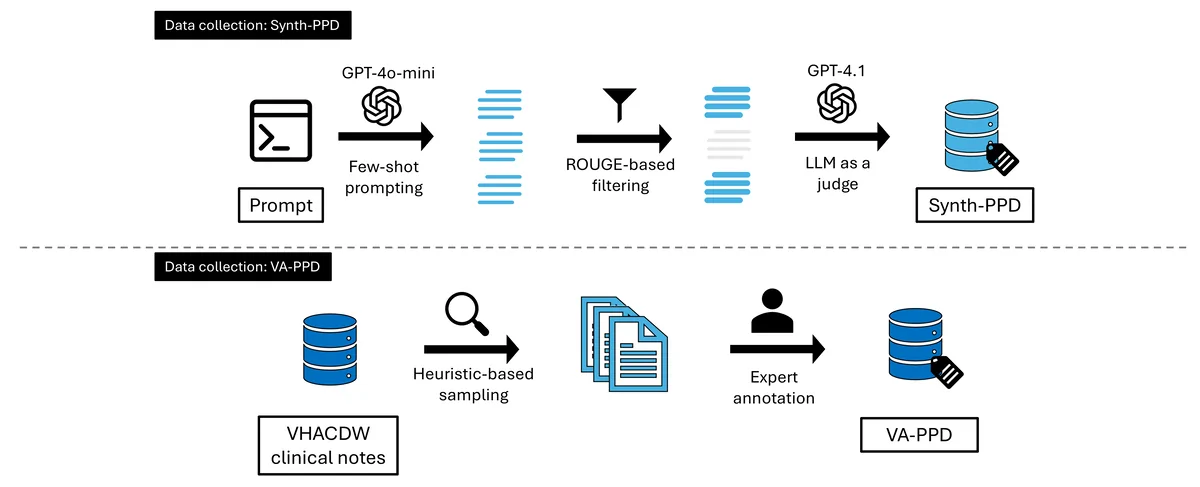
Data collection pipeline. LLM: large language model; ROUGE: Recall-Oriented Understudy for Gisting Evaluation; VHACDW: Veterans Health Administration Corporate Data Warehouse.

### Real-World Dataset (VA-PPD) Collection

For external evaluation, we used clinical notes from the US Veterans Health Administration Corporate Data Warehouse. This dataset, referred to as VA-PPD, consisted of 293 clinical notes manually annotated by domain experts for mentions of parole or probation.

To ensure adequate representation of relevant cases, at least half of the notes were selected using a combination of an existing SBDH detection system [22] and keyword-based filtering. Specifically, the SBDH system identified notes containing mentions of legal problems, after which keyword searches for words—*parole* and *probation*—were applied.

All selected notes were independently annotated by 2 experts following the same guidelines used for Synth-PPD. For VA-PPD, we adopted a simplified labeling scheme and retained only examples with *presence*=yes. The final dataset included 92 notes labeled as *parole*, 84 labeled as *probation*, and 117 labeled as *unclear* or *neither*.

### PPD Framework Design

All supervised PPD systems were trained exclusively on Synth-PPD without any human-annotated training data. During training of transformer encoder and LLM systems, we formulated the PPD task as a multitask, multiple-choice question answering problem.

In the unsupervised setting, we used a publicly available LLM with task-specific prompts to elicit predictions on all attributes (*label*, *presence*, and *period*). We explored 2 ICL strategies: few-shot prompting [23] and chain of thought (COT) prompting [24], as well as their combination. Few-shot examples were selected from the Synth-PPD training set using a verbalization-based uncertainty estimation method [25] wherein the LLM was prompted to output confidence scores for its predictions. Few-shot demonstrations were selected from the pool of examples associated with the lowest confidence under the assumption that uncertainty-driven example selection can improve performance.

In the supervised setting, we applied prompt-based fine-tuning [26,27], training models to map a masked token to one of the multiple-choice question answering choices. The unsupervised and supervised PPD frameworks are illustrated in Figures 2-4.

**Figure 2:**
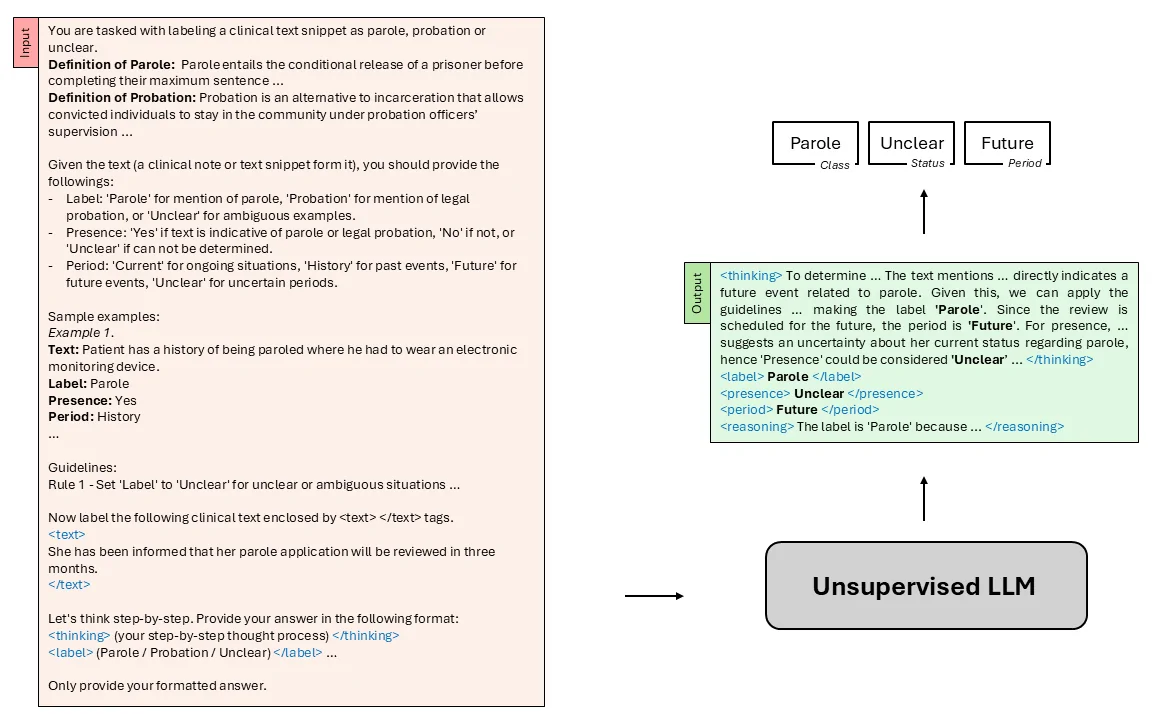
Parole and probation detection pipeline under the unsupervised setting. LLM: large language model.

**Figure 3:**
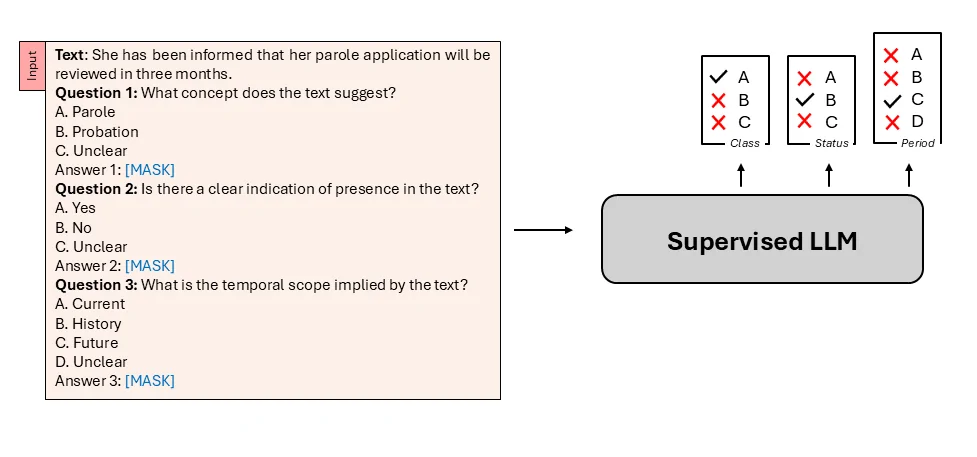
Input preprocessing and task formulation under the supervised setting. LLM: large language model.

**Figure 4:**
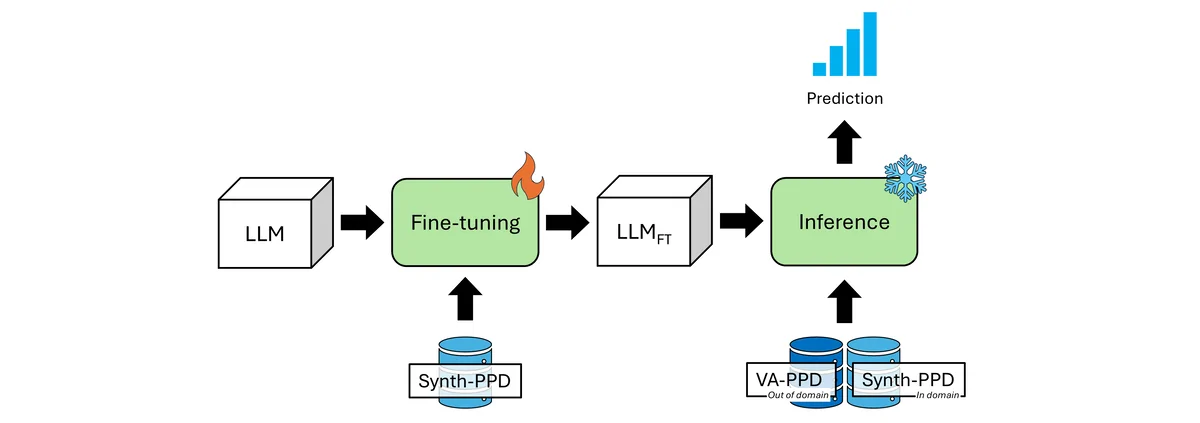
Parole and probation detection pipeline under the supervised setting. FT: fine-tuning; LLM: large language model.

### Evaluation Protocol

All systems were evaluated on the in-domain, expert-reviewed Synth-PPD test set and out-of-domain, real-world VA-PPD dataset to assess cross-domain generalization. The PPD task was defined as identifying whether a record indicates current parole, current probation, or *unclear* or *neither* (ie, *presence*=yes and *period*=current), resulting in a multi-class classification problem.

Unlike Synth-PPD examples, which contain one to a few sentences, VA-PPD examples consist of full clinical notes. To align evaluation conditions for supervised models, each note was segmented into overlapping 3-sentence paragraphs with a 1-sentence overlap, yielding 5341 segments. Paragraph-level predictions were aggregated to produce note-level classifications. Performance was evaluated using macroprecision, macrorecall, and macro–*F*_1_-score.

### Baselines and Experimental Settings

We evaluated both unsupervised and supervised baselines. The basic rule-based (keywords) baseline classified notes as parole if the word *parole* was present, as probation if the word *probation* was present, and as *unclear* or *neither* otherwise. A second rule-based baseline was implemented using similar keyword matching for parole and probation terms combined with a simple negation detection mechanism. Negation cues within a fixed token window preceding the keyword were used to identify and exclude negated mentions. We also included majority class baseline. As an unsupervised LLM baseline, we used Llama 3.3-70B-Instruct (Meta AI) [28]. For supervised learning, we considered 3 classes of models—traditional ML models such as logistic regression, random forest, and gradient boosting; transformer encoders such as RoBERTa-base [29] and clinical RoBERTa-base [30]; and LLMs such as Llama 3.1-8B (Meta AI) [31] and Qwen2.5-7B (Alibaba Cloud) [32]. To assess model stability, for traditional ML models, we performed stratified k-fold cross-validation on the training data (*k*=10), whereas for others, we fine-tuned them with 3 different seeds. Hyperparameters were optimized using the Synth-PPD validation set. GPT model parameters, all prompts used in our experiments, and annotation guidelines for Synth-PPD are provided in Multimedia Appendix 1.

All systems were evaluated on both Synth-PPD (in domain) and VA-PPD (out of domain). Interannotator agreement was 91% for Synth-PPD and 84.21% for VA-PPD, calculated as the proportion of examples for which both annotators agreed on *label*, *presence*, and *period*, with disagreements resolved through expert adjudication. Moreover, to independently assess the quality of the Synth-PPD dataset, we compared expert-reviewed labels of the test split with the LLM-generated labels, leading to a human-LLM agreement of 90.05%. *P* values were calculated using bootstrap testing. Dataset statistics are reported in Table 1. All analyses followed the TRIPOD+AI (Transparent Reporting of a Multivariable Prediction Model for Individual Prognosis or Diagnosis–Artificial Intelligence) guidelines [33]. Experiments were conducted in Python (version 3.12.2; Python Software Foundation) using PyTorch (version 2.2.2; Meta AI) [34].

**Table 1:** Dataset statistics^a^.

		Synth-PPD (n=7579)	VA-PPD (n=293)
**Text characteristics**			
	Word count, mean (SD)	15.23 (2.32)	728.71 (629.01)
	Word count, median (IQR)	15 (14-17)	557 (219-1052)
	Sentence count, mean (SD)	1 (0.01)	36.17 (32.45)
	Sentence count, median (IQR)	1 (1-1)	26 (12-50)
**Class distribution, n (%)**			
	Parole	1589 (21)	92 (31.4)
	Probation	2920 (38.5)	84 (28.7)
	Unclear or neither	3070 (40.5)	117 (39.9)
Negated (presence=no), n (%)		1900 (25.1)	0 (0)
Source		GPT-4o-mini	VHA-CDW^b^
Verifier		GPT-4.1	—^c^
Labeler		GPT-4o-mini+human	Human
Annotation rationale		Present	Absent
Availability		Public	Private

^a^Segment-level text characteristics are reported in Table S1 in Multimedia Appendix 1.

^b^VHACDW: Veterans Health Administration Corporate Data Warehouse.

^c^Verification is not applicable to VA-PPD as it consists of real-world notes.

### Ethical Considerations

This study was reviewed and approved by the institutional review board of the VA Bedford Healthcare System. Informed consent was waived by the institutional review board because the study involved secondary analysis of existing clinical data and did not involve direct participant recruitment or interaction. There was no compensation provided to participants, and all medical records data were accessed on a secure server behind the VA’s firewall.

## Results

### Data Analysis

At the model input level, Synth-PPD examples were shorter than VA-PPD segments, with mean token counts of 15.23 (SD 2.32) vs 58.28 (SD 69.33, standardized mean difference −0.878) and mean character counts of 94.23 (SD 13.22) vs 403.99 (SD 573.66, standardized mean difference −0.763; Table S1 in Multimedia Appendix 1). These differences reflect the different contextual granularity of the 2 datasets: Synth-PPD examples were generated as individual examples, whereas VA-PPD notes were segmented into overlapping 3-sentence model input windows for evaluation, with predictions subsequently aggregated to the note level. In Synth-PPD, 25.1% (1900/7579) of examples were explicitly negated (*presence*=no), whereas VA-PPD contained no negated examples. Class distributions are reported in Table 1.

### Overall Performance

We report the results in Table 2. Across all model classes, performance on the synthetic Synth-PPD dataset was substantially higher than that on the real-world VA-PPD dataset, reflecting the presence of domain shift between synthetic and real-world clinical text. Rule-based systems achieved moderate performance, with the keyword-based baseline reaching a 50.61% macro–*F*_1_-score on Synth-PPD and a 76.51% macro–*F*_1_-score on VA-PPD. Incorporating negation handling substantially improved performance on Synth-PPD (60.72% macro–*F*_1_-score) but yielded only marginal gains on VA-PPD (77.55% macro–*F*_1_-score).

**Table 2:** Performance comparison of different parole and probation detection systems on in-domain (Synth-PPD) and out-of-domain (VA-PPD) datasets.

Systems				In domain (Synth-PPD)							Out of domain (VA-PPD)				
				Macroprecision (%)		Macrorecall (%)		Macro–*F*_1_-score (%)		Macroprecision (%)			Macrorecall (%)		Macro–*F*_1_-score (%)
**Unsupervised**															
	**Rule based**														
		Majority class baseline	12.44		33.33		18.11		13.31			33.33		19.02	
		Keyword based	74.24		66.84		50.61		81.63			80.97		76.51	
		Keyword+negation rules	68.43		70.02		60.72		80.78			81.24		77.55	
	**LLM^a^**														
		Zero shot, mean (SD)	79.34 (0.12)		81.20 (0.09)		78.33 (0.10)		83.32 (0.14)			83.35 (0.16)		80.10 (0.17)	
		Few shot, mean (SD)	90.09 (0.09)		90.79 (0.11)		90.33 (0.11)		87.16 (0.14)			88.43 (0.13)		87.04 (0.16)	
		COT^b^, mean (SD)	82.56 (0.43)		82.63 (0.47)		78.98 (0.60)		83.62 (0.26)			83.53 (0.07)		80.40 (0.21)	
		COT+few shot, mean (SD)	91.22 (0.32)		92.57 (0.23)		91.72 (0.30)		87.02 (1.20)			88.22 (1.14)		87.22 (1.20)	
**Supervised**															
	**Traditional ML^c^**														
		Logistic regression, mean (SD)	89.70 (0.32)		90.09 (0.28)		89.88 (0.29)		70.01 (1.28)			62.82 (1.11)		55.70 (1.21)	
		Random forest, mean (SD)	88.71 (0.32)		88.73 (0.29)		88.72 (0.29)		57.85 (3.36)			55.63 (4.79)		54.47 (5.38)	
		Gradient boosting, mean (SD)	90.03 (0.31)		89.70 (0.27)		89.86 (0.28)		47.32 (4.29)			45.91 (3.70)		35.67 (2.95)	
	**Transformer encoders**														
		RoBERTa-base, mean (SD)	95.47 (0.23)		95.55 (0.38)		95.48 (0.32)		40.39 (2.34)			35.31 (2.09)		29.85 (0.55)	
		Clinical RoBERTa-base, mean (SD)	96.07 (0.14)		96.02 (0.07)		96.03 (0.10)		42.52 (5.30)			37.77 (2.60)		29.96 (1.17)	
	**LLM**														
		Qwen2.5-7B, mean (SD)	95.96 (0.41)		95.94 (0.37)		95.91 (0.39)		79.79 (2.29)			79.69 (2.62)		79.38 (2.66)	
		Llama 3.1-8B, mean (SD)	96.12 (0.62)		96.07 (0.82)		96.07 (0.75)		81.47 (1.05)			81.82 (1.02)		81.43 (1.16)	

^a^LLM: large language model.

^b^COT: chain of thought.

^c^ML: machine learning.

All traditional ML models demonstrated strong in-domain performance, with macro–*F*_1_-scores approaching 90% on Synth-PPD. However, these models exhibited substantial degradation when evaluated on VA-PPD, with the biggest performance observed for the gradient boosting model at a 35.67% macro–*F*_1_-score and the lowest performance observed for logistic regression at a 55.70% macro–*F*_1_-score. Transformer encoder models showed a similar pattern, achieving the highest in-domain performance (up to 96.03% macro–*F*_1_-score for clinical RoBERTa), matching even 60.9 to 64.2 times larger LLMsbut performing poorly out of domain, underperforming (approximately 30% macro–*F*_1_-score) both the best rule-based system and traditional ML models, indicating limited robustness to distribution shift despite strong in-domain performance.

All LLM-based systems substantially outperformed other baselines in out-of-domain evaluations while exceeding or matching the best-performing baseline in domain. For instance, in the supervised setting, Llama 3.1-8B achieved a macro–*F*_1_-score of 96.07% on Synth-PPD, exceeding the best rule-based system (keyword-based with negation rules) by 58.22% (*P*<.001) and the best traditional ML model (logistic regression) by 6.89%. On VA-PPD, the same model improved performance by 5% relative to the best rule-based baseline (*P*<.001), by 46.19% relative to the best traditional ML model (logistic regression), and by 171.80% relative to the best transformer encoder model (clinical RoBERTa). Similar to traditional ML models and transformer encoders, all LLMs experienced a notable performance decline under out-of-domain evaluation. The best unsupervised system, Llama 3.3-70B-Instruct with examples from Synth-PPD (COT+few shot), achieved a macro–*F*_1_-score of 87.22% in the out-of-domain setting, surpassing its zero-shot setting by 8.89%. The same system also outperformed the best rule-based, traditional ML, transformer encoder, and supervised LLM baselines by 12.47%, 56.59%, 191.12%, and 7.11%, respectively.

Comparisons between supervised and unsupervised approaches reveal complementary strengths. Supervised models dominated in-domain performance, whereas unsupervised methods achieved superior results in the out-of-domain setting. Notably, the best supervised model, Llama 3.1-8B, outperformed the approximately 9 times larger Llama 3.3-70B-Instruct model in the zero-shot setting by 22.65% (*P*<.001) in domain; out of domain, Llama 3.1-8B achieved a numerically higher macro–*F*_1_-score by 1.66%, although this was not statistically significant (*P*=.08). However, supervised models did not consistently surpass the strongest unsupervised systems out of domain. Among unsupervised approaches, few-shot prompting consistently outperformed zero-shot and COT-only variants across both domains. In particular, the COT with few-shot configuration achieved the highest out-of-domain performance among unsupervised methods, exceeding the best supervised model, Llama 3.1-8B, by 7.11% (87.22% vs 81.43%; *P*<.001) without requiring expert-labeled training data. Unsupervised NLP systems also exhibited greater performance stability across domains.

### Impact of Data Size

To examine the impact of data scale on real-world deployment, we focused on the LLM baselines and out-of-domain VA-PPD dataset (Table 3). In the unsupervised setting, increasing the number of few-shot examples led to substantial gains over zero-shot prompting, with macro–*F*_1_-score improving consistently up to 30 shot (from 80.10% to 87.04% without COT and from 80.40% to 87.22% with COT), followed by no improvement beyond this point. In the supervised setting, increasing the amount of labeled training data improved performance across all NLP systems and enhanced their stability. Fine-tuning on the full Synth-PPD dataset consistently yielded higher macro–*F*_1_-scores than training on half of the data for both Qwen2.5-7B and Llama 3.1-8B, although the magnitude and statistical significance of the improvement differed by model. For Llama 3.1-8B, performance improved by 31.06% (81.43% vs 62.13%; *P*<.001), whereas the 2.56% (79.38% vs 77.40; *P*=.28) improvement for Qwen2.5-7B was not statistically significant.

**Table 3:** Performance trend on VA-PPD (out of domain) with change in data size^a^.

Systems				Macroprecision (%), mean (SD)		Macrorecall (%), mean (SD)		Macro–*F*_1_-score (%), mean (SD)
**Unsupervised**								
	**Llama 3.3-70B-Instruct**							
		Zero shot	83.32 (0.14)		83.35 (0.16)		80.10 (0.17)	
		Few shot—*k*=15	84.55 (0.11)		85.49 (0.13)		83.10 (0.17)	
		Few shot—*k*=30	87.16 (0.14)		88.43 (0.13)		87.04 (0.16)	
		Few shot—*k*=60	86.02 (0.20)		87.28 (0.22)		85.90 (0.17)	
		COT^b^	83.62 (0.26)		83.53 (0.07)		80.40 (0.21)	
		COT+few shot—*k*=15	84.57 (0.36)		85.49 (0.24)		83.25 (0.30)	
		COT+few shot—*k*=30	87.02 (1.20)		88.22 (1.14)		87.22 (1.20)	
		COT+few shot—*k*=60	86.05 (0.68)		87.24 (0.67)		86.18 (0.75)	
**Supervised**								
	**Qwen2.5-7B**							
		SFT-Synth-PPD-half	78.44 (3.13)		77.83 (4.46)		77.40 (4.51)	
		SFT-Synth-PPD-full	79.79 (2.29)		79.69 (2.62)		79.38 (2.66)	
	**Llama 3.1-8B**							
		SFT-Synth-PPD-half	70.59 (1.19)		63.28 (4.65)		62.13 (5.18)	
		SFT-Synth-PPD-full	81.47 (1.05)		81.82 (1.02)		81.43 (1.16)	

^a^SFT-Synth-PPD-half and SFT-Synth-PPD-full denote supervised models fine-tuned on 50% and 100% of the Synth-PPD training set, respectively.

^b^COT: chain of thought.

## Discussion

### Principal Findings

We present a synthetic-only training paradigm demonstrating that LLM-generated synthetic data can be effectively leveraged to identify indicators of criminal justice involvement—specifically parole and probation—in clinical text. To the best of our knowledge, this is the first study to examine the use of LLM-generated synthetic data for the PPD task in the clinical domain. By validating our approach on real-world data from the VA health care system, we showed that synthetic data can serve as a viable alternative when access to real-world data are constrained. Moreover, by systematically evaluating multiple model classes, we provide new insights into how different approaches leverage synthetic data and generalize across distributions in the context of PPD. The proposed framework also helps mitigate challenges inherent to clinical data, including privacy concerns, limited computational resources, and the high cost of expert annotation.

One notable finding is the dataset-dependent impact of contextual linguistic features, particularly negation. The basic rule-based (keywords only) baseline achieved comparatively stronger performance on VA-PPD than on Synth-PPD, potentially reflecting the relative simplicity of many real-world PPD instances, pronounced more through the difference in the distribution of negated expressions between synthetic and real-world data. For example, clinicians and social workers are less likely to discuss legal issues unless there is a probable cause for them to believe that the information is pertinent to patient care or the patient is confirmed to undergo legal problems. In our setup, VA-PPD was constructed using only examples with *presence*=yes, and therefore, it contains no explicitly negated PPD examples, whereas 25.1% (1900/7579) of the Synth-PPD examples were explicitly negated (*presence*=no). This difference is consistent with the larger impact of incorporating negation handling into the rule-based system, which improved the macro–*F*_1_-score by 19.98% on Synth-PPD compared with 1.36% on VA-PPD. This substantially larger improvement on Synth-PPD suggests that the explicit inclusion of negated examples contributes to the greater sensitivity of rule-based performance to negation in the synthetic dataset. These findings highlight how synthetic data design choices, such as the inclusion of negation, can substantially alter task characteristics and model behavior. However, the rule-based approach exhibits important limitations: it cannot reliably distinguish current from historical mentions of parole or probation or disambiguate nonlegal uses of related terms (eg, “academic probation”). It may also incorrectly attribute legal status described for relatives or acquaintances to the patient. Such distinctions are difficult to encode through keyword-based heuristics but can be more effectively captured by LLMs trained on synthetic data with diverse contextual attributes, leading to improved robustness.

Across experiments, LLMs emerged as the best-performing systems in both in-domain and out-of-domain settings. Specifically, our study demonstrates that LLMs with Synth-PPD can effectively identify parole and probation status in real-world clinical notes, outperforming rule-based systems, traditional ML models, and transformer encoders under domain shift. For example, on the real-world VA-PPD dataset, both unsupervised and supervised LLM-based systems substantially outperformed the rule-based, traditional ML, and transformer encoder baselines. Supervised models achieved the strongest in-domain performance but exhibited reduced advantages under out-of-domain evaluation. In contrast, unsupervised LLM-based approaches demonstrated more stable performance across domains, particularly when augmented with few-shot examples from Synth-PPD. These findings suggest that supervised learning on synthetic data may have limited cross-domain scalability relative to unsupervised NLP approaches. Notably, the COT plus few-shot configuration yielded the strongest out-of-domain performance among unsupervised methods, surpassing supervised models without requiring labeled training data. At the same time, supervised Llama 3.1-8B outperformed the approximately 9 times larger Llama 3.3-70B-Instruct in the zero-shot setting for both in-domain and out-of-domain evaluations. Together, these results highlight a trade-off: supervised models excel under strong domain alignment, whereas unsupervised LLM-based methods offer greater robustness and adaptability under domain shift.

Beyond performance gain, this study provides empirical evidence that model architecture plays a critical role in how synthetic data are used, with LLMs demonstrating fundamentally different cross-domain performance compared to traditional and encoder-based models. Traditional ML models and transformer encoders achieved strong in-domain performance but experienced substantial degradation when evaluated on the real-world VA-PPD dataset. Surprisingly, transformer encoders performed worse than traditional ML models in this out-of-domain setting despite their superior in-domain performance. One possible explanation is that encoder-based models may be more sensitive to distributional patterns of the synthetic data that differ from naturally occurring clinical documentation, possibly contributing to the observed performance degradation under domain shift. In contrast, LLM-based approaches demonstrated strong robustness to domain shift. Both unsupervised and supervised LLMs maintained high performance when evaluated on VA-PPD, indicating that these models can leverage broader semantic and contextual knowledge beyond surface lexical patterns and distribution-specific signals. This superior cross-domain performance of LLMs may stem from their large-scale pretraining, which enables them to capture more abstract representations of language and apply them across domains.

We further observed that increasing the number of few-shot examples from Synth-PPD improved performance in the unsupervised setting, primarily through significant reductions in false positives. For example, in the COT-based few-shot configuration, adding 30 examples increased precision for parole by 5.71% (83.02% to 87.76%) and for probation by 22.06% (68.85% to 84.04%; Figure 5A and Figure 5B). Consistent with prior work on ICL [35,36], performance eventually plateaued, after which additional examples yielded no further gains (Table 3). In the supervised setting, larger volumes of synthetic training data improved both performance and stability. For Llama 3.1-8B evaluated on VA-PPD, doubling synthetic training data improved precision for parole by 49.52% (49.70% to 74.31%) and for probation by 7.20% (77.36% to 82.93%; Figure 5C and Figure 5D), suggesting enhanced task understanding and reduced classification errors. These results support our earlier observations that LLMs trained on synthetic data better capture negation, temporality, and contextual cues, thereby reducing false positives and improving accuracy relative to rule-based approaches. The effectiveness of synthetic data in the PPD task may partly stem from the relatively explicit lexical markers associated with parole and probation status. Unlike more abstract clinical phenomena, legal supervision status is often described using specific terminology, which can be captured effectively through prompt-based synthetic generation.

**Figure 5:**
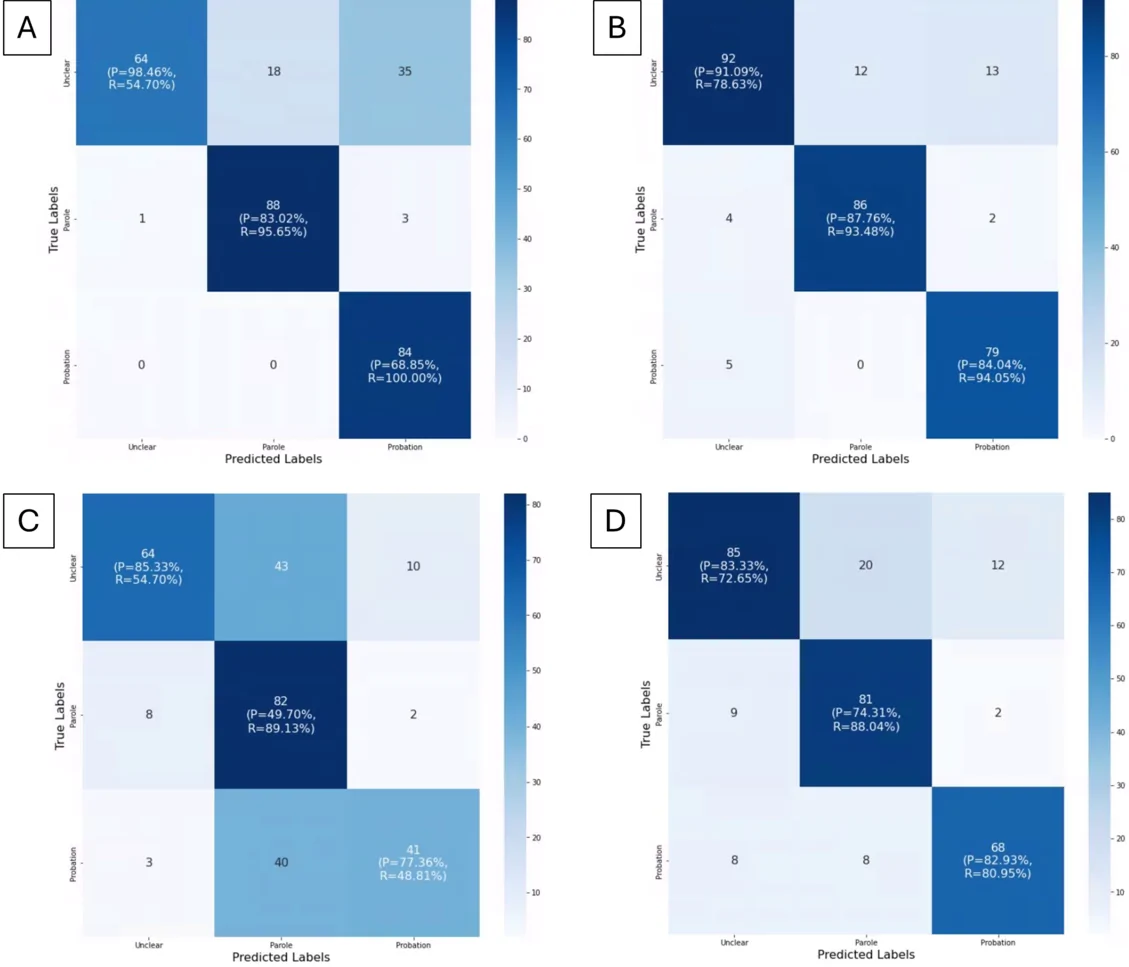
Confusion matrices on the VA-PPD dataset: (A) chain of thought (COT; Llama 3.3-70B-Instruct), (B) COT+30 shot (Llama 3.3-70B-Instruct), (C) SFT-Synth-PPD-half (Llama 3.1-8B), and (D) SFT-Synth-PPD-full (Llama 3.1-8B). P: precision; PPD: parole and probation detection; R: recall.

This work introduces a potential pathway toward automated detection and monitoring of a sensitive legal indicator—the presence of parole or probation—in clinical documentation. Recent studies have shown that synthetic data can substantially improve NLP systems on real-world clinical tasks such as identifying eviction [13] and other SBDH [12,22] factors, clinical note generation [15], etc. Compared to past work, our contribution is twofold. First, despite its recognized relevance to the VA [8], using unstructured clinical text for PPD among veterans remains underexplored. We bridged that gap by developing Synth-PPD and targeting the PPD task for clinical text. Second, unlike most prior studies that emphasize in-domain gains, we provided a systematic cross-domain analysis across model classes without leveraging explicit domain adaptation techniques. Our proposed framework offers a scalable and privacy-preserving approach for extracting such information without expert supervision. Similar to existing studies that used automatic SBDH extraction frameworks [22,37], scaling our PPD framework could also support epidemiological analyses of parole and probation among veterans, helping address gaps in understanding criminal justice involvement in this population. The findings from this work may also inform guidelines and best practices for documenting criminal justice involvement within the VA health care system.

Our findings also contribute to the broader problem of domain adaptation in clinical ML. Our corpus-level analysis (Table 1) identified measurable differences in text characteristics between Synth-PPD and VA-PPD. VA-PPD examples, even when segmented, were longer than Synth-PPD examples in terms of token and character counts, reflecting differences in the contextual granularity of the 2 datasets. Despite these differences, models trained on Synth-PPD demonstrated effective transfer to VA-PPD, suggesting that the synthetic examples captured sufficient task-relevant information for cross-domain PPD task. This also highlights the potential for synthetic data to serve as a viable proxy for sensitive clinical text in other tasks. At the same time, deploying such a framework necessitates careful consideration of its ethical and clinical implications for veterans, patients, and service provision [38-40]. Information regarding probation or parole status may be stigmatizing, and awareness of a veteran’s status may not be clinically relevant for many clinicians. Inappropriate sharing or use of this information could erode patient trust and adversely affect care. Additionally, misclassification of probation or parole status could negatively influence patients’ access to services. These considerations underscore the need to clearly define the contexts and safeguards under which a PPD framework could be responsibly applied in research or clinical settings, which should be addressed in future work.

### Limitations

Several limitations warrant consideration in this study. First, validation was conducted using a single real-world dataset (VA-PPD) from the VA health care system, limiting representativeness of the general US population and the broader veteran population. In addition, VA-PPD comprised 293 notes, limiting our ability to assess heterogeneity across health care systems, sites, services, or periods. Moreover, VA-PPD was derived from SBDH screening and keyword-based filtering. Although this approach enabled construction of a feasible evaluation corpus enriched for the target concepts, it may not reflect the full linguistic diversity of such documentation in general clinical notes. Larger, independently sampled, and multi-institutional evaluations are needed to assess generalizability. Second, synthetic datasets such as Synth-PPD may introduce biases inherited from LLMs [41-43], which may be particularly consequential when representing sensitive justice-related information, leading models to rely on patterns that are problematic and less prevalent in real clinical documentation. Moreover, Synth-PPD may not fully capture the linguistic diversity and complexity of real-world clinical documentation. Future work should explore more diverse data generation strategies to mitigate such biases, including prompt diversification and human-in-the-loop filtering. Third, although LLMs showed substantial improvements when trained on synthetic data, there remains room for enhancement. Future work will explore alternative strategies for few-shot example selection and prompting in unsupervised settings, such as self-consistency [44], self-verification [45], and multi-agent frameworks [46]. Fourth, evaluation was restricted to a limited set of LLM baselines due to privacy and resource constraints; future studies will assess additional HIPAA (Health Insurance Portability and Accountability Act)-compliant LLMs such as GPT-5 [47]. Finally, transformer encoder models—which underperform substantially in the cross-domain setting—may benefit from explicit domain adaptation strategies such as prompt tuning, P-tuning, lightweight adapter modules, and adversarial training. Future work will systematically evaluate such methods to disentangle whether the observed performance gap is due to inherent model limitations or the absence of adaptation mechanisms.

### Conclusions

In summary, this is the first study to introduce a scalable and privacy-preserving framework for detecting parole and probation mentions in clinical notes using LLM-generated synthetic data, reducing reliance on privacy-restricted, expert-annotated clinical data. Synth-PPD enables the development of NLP systems without expert annotation and demonstrates successful cross-domain transfer to real-world VA data. Future work should pursue larger, independently sampled, and multi-institutional validation; prospective evaluation in clinical workflows; assessment of demographic fairness; and comparison of synthetic data development with conventional human annotation workflows. By addressing key challenges in health care informatics—including data scarcity, privacy constraints, and annotation costs—this work advances clinically relevant information extraction in the underexplored area of criminal justice involvement among veterans and lays a foundation for future methodological and epidemiological research.

## Appendix

Multimedia Appendix 1 Model parameters, used prompts, annotation guidelines, and data stats.

## Abbreviations

COT: chain of thought
HIPAA: Health Insurance Portability and Accountability Act
ICL: in-context learning
LLM: large language model
ML: machine learning
NLP: natural language processing
PPD: parole and probation detection
ROUGE: Recall-Oriented Understudy for Gisting Evaluation
SBDH: social and behavioral determinants of health
TRIPOD+AI: Transparent Reporting of a Multivariable Prediction Model for Individual Prognosis or Diagnosis–Artificial Intelligence
VA: United States Department of Veterans Affairs

## Data Availability

Synth-PPD is publicly available at Hugging Face [48]. The VA-PPD dataset is available under restricted access due to veteran privacy protection and data security laws. Access can be obtained via relevant approvals through the US Department of Veterans Affairs Informatics and Computing Infrastructure (contact: VINCI@va.gov). Individuals who wish to use these data for research purposes must fulfill the research credentialing requirements as outlined by the US Department of Veterans Affairs Office of Research and Development. Our codes are also publicly available at Github [49].
