# Supplementary material for: Large Language Model–Generated Synthetic Data for Parole and Probation Detection in Clinical Notes: Development and Evaluation Study
Source: JMIR AI. 2026 Oct 19;5:e104586. doi: 10.2196/104586 (PMC13728768; doi:10.2196/104586)
Supplement: Supplementary file 1 [file ai_v5i1e104586_app1.docx]

Model parameters, used prompts, annotation guidelines and data stats

# Multimedia Appendix

## 1. Model Parameters

|  | GPT-4o-mini | GPT-4.1 |
| --- | --- | --- |
| Model version | gpt-4o-mini-2024-07-18 | gpt-4.1-2025-04-14 |
| Generation date | September 2025 | September 2025 |
| Temperature | 0.8 | 0.8 |
| Top P | 1 | 1 |
| Prompt | Multimedia Appendix 2 | Multimedia Appendix 3 |

## 2. Prompt for GPT-4o-mini

*You are tasked with generating synthetic data for a project focused on the intersection of legal issues (specifically parole and probation) and clinical text. Use the following definitions to guide your annotations.*

*Definition of Parole: Parole entails the conditional release of a prisoner before completing their maximum sentence, granted by a parole board or similar authority. This release is contingent on demonstrated rehabilitation and the ability to reintegrate into society without posing a threat. The primary objective is to provide supervision and support, aiding offenders in a successful transition back into the community. Parolees serve their time in the community and can be returned to prison for violating any of the conditions of parole imposed by the Board of Parole Commissioners.*

*Definition of Probation: Probation is an alternative to incarceration that allows convicted individuals to stay in the community under probation officers’ supervision. Unlike parole, probation is often part of the original sentence and is not granted after serving time in prison. It serves purposes such as punishment, rehabilitation, and community protection, giving offenders a chance to address their behavior while remaining part of the community. Probation is very similar to parole except that probationers have not served prison time on their suspended sentence and the sentencing judge maintains jurisdiction over the probationer.*

*Generate examples that mimic snippets from patients' electronic health records (EHR). Each example should include:*

*- Text: A brief clinical note mentioning parole or probation.*

*- Label: 'Parole' for mention of parole, 'Probation' for mention of legal probation, or 'Unclear' for ambiguous examples.*

*- Presence: 'Yes' if 'Text' is indicative of parole or legal probation, 'No' if not, or 'Unclear' if can not be determined.*

*- Period: 'Current' for ongoing situations, 'History' for past events, 'Future' for future events, 'Unclear' for uncertain periods.*

*Sample examples:*

*Example 1.*

*Text: Patient has a history of being paroled where he had to wear an electronic monitoring device.*

*Label: Parole*

*Presence: Yes*

*Period: History*

*Example 2.*

*Text: She had to stay within the state boundaries unless given permission to travel.*

*Label: Probation*

*Presence: Yes*

*Period: Current*

*Example 3.*

*Text: He states they are rude to him and it seems more like a parole office than a doctor's office.*

*Label: Parole.*

*Presence: No.*

*Period: Current.*

*...*

*Example 10.*

*Text: Due to violation of her probation rules, the patient ended up serving a short time in prison.*

*Label: Probation*

*Presence: Yes*

*Period: History*

*Step-by-step instructions:*

*Step 1 - For each example, generate the 'Text' field first.*

*Step 2 - Next, analyze the text step-by-step and provide rationales for appropriate label, presence, and period values to the example 'Text'. Provide this justification as 'Reasoning'.*

*Step 3 - Finally, according to the 'Reasoning', provide the values for 'Label', 'Presence', and 'Period'.*

*Guidelines:*

*Rule 1 - Each example 'Text' should emulate a text snippet from a patient's EHRs with no more than three sentences.*

*Rule 2 - 'Reasoning' should be self-sufficient and brief with no more than three sentences.*

*Rule 3 - You must make the examples (especially 'Text' and 'Reasoning') as diverse as possible, both syntactically and semantically. Do not start examples with the same pattern.*

*Rule 4 - Set 'Label' to 'Unclear' for unclear or ambiguous situations. Examples:*

*- Example 1.*

*Text: The review of the patient's file indicates no history of parole or probation.*

*Label: Unclear*

*Presence: No*

*Period: History*

*- Example 2.*

*Text: The patient recalls wearing an electronic monitoring device for six months ten years ago, as required by his then-release terms.*

*Label: Unclear*

*Presence: Yes*

*Period: History*

*- Example 3.*

*Text: Veteran has been under strict supervision for two months.*

*Label: Unclear*

*Presence: Yes*

*Period: Current*

*Rule 5 - When 'Period' is 'Future', determine 'Presence' carefully. For instance, mention of an upcoming parole hearing does not guarantee parole and hence, 'Label'='Parole', 'Presence'='Unclear', 'Period'='Future'. However, for clear indications such as 'will be granted parole in two weeks', 'Label'='Parole', 'Presence'='Yes', 'Period'='Future'.*

*Rule 6 - Make a balance by considering different combinations of 'Label', 'Presence', and 'Period' values. Remember to limit 'Label', 'Presence', and 'Period' values to the categories described before.*

*Rule 7 - Always generate some negative examples i.e., examples with 'Presence'='No'. The 'Label' can be either 'Parole', 'Probation' or 'Unclear', depending on 'Text'.*

*Rule 7.1 - Examples with negative indications of parole or probation, e.g., 'no parole', 'pt was denied probation' etc.*

*Rule 7.2 - Examples with non-legal probation, e.g., employment probation, school disciplinary probation, sports team probation, etc. ('Label'='Probation')*

*Rule 7.3 - Examples with parole/probation related to the patient's family, relatives, or friends, but not the patient themselves. ('Label'='Parole'/'Probation')*

*Rule 8 - Do not copy any example from the sample examples.*

*Now, generate 20 examples that strictly meet the instructions and guidelines mentioned above. Format examples as a valid JSON with the following structure:*

*{'examples':*

*[*

*{*

*'Text':...,*

*'Reasoning':...,*

*'Label':...,*

*'Presence':...,*

*'Period':...,*

*},*

*{*

*'Text':...,*

*'Reasoning':...,*

*'Label':...,*

*'Presence':...,*

*'Period':...,*

*},*

*...*

*]*

*}*

*Only provide the JSON-formatted response.*

## 3. Prompt for GPT-4.1

*You are tasked with evaluating synthetic examples on the intersection of legal issues (specifically parole and probation) and clinical text. Use the following definitions to guide your annotations.*

*Definition of Parole: Parole entails the conditional release of a prisoner before completing their maximum sentence, granted by a parole board or similar authority. This release is contingent on demonstrated rehabilitation and the ability to reintegrate into society without posing a threat. The primary objective is to provide supervision and support, aiding offenders in a successful transition back into the community. Parolees serve their time in the community and can be returned to prison for violating any of the conditions of parole imposed by the Board of Parole Commissioners.*

*Definition of Probation: Probation is an alternative to incarceration that allows convicted individuals to stay in the community under probation officers’ supervision. Unlike parole, probation is often part of the original sentence and is not granted after serving time in prison. It serves purposes such as punishment, rehabilitation, and community protection, giving offenders a chance to address their behavior while remaining part of the community. Probation is very similar to parole except that probationers have not served prison time on their suspended sentence and the sentencing judge maintains jurisdiction over the probationer.*

*Each example includes five fields:*

*- Text: A brief clinical note mentioning parole or probation.*

*- Label: Accepted values are:*

*- 'Parole' for mention of parole,*

*- 'Probation' for mention of legal probation, or*

*- 'Unclear' for ambiguous examples.*

*- Presence: Accepted values are:*

*- 'Yes' if 'Text' is indicative of parole or legal probation,*

*- 'No' if not, or*

*- 'Unclear' if can not be determined.*

*- Period: Accepted values are:*

*- 'Current' for ongoing situations,*

*- 'History' for past events,*

*- 'Future' for future events, or*

*- 'Unclear' for uncertain periods.*

*- Reasoning: A brief reasoning for the values of 'Label' , 'Presence' and 'Period'.*

*Guidelines:*

*Rule 1 - 'Label' should be 'Unclear' for unclear or ambiguous situations. 'Presence' and 'Period' values should depend on the context.*

*- Example 1. (clear indication of no parole or probation in the past but not specific)*

*Text: The review of the patient's file indicates no history of parole or probation.*

*Label: Unclear*

*Presence: No*

*Period: History*

*- Example 2. (clear indication of parole or probation in the past but not specific)*

*Text: The patient recalls wearing an electronic monitoring device for six months ten years ago, as required by his then-release terms.*

*Label: Unclear*

*Presence: Yes*

*Period: History*

*- Example 3. (clear indication of current parole or probation but not specific)*

*Text: Veteran has been under strict supervision for two months.*

*Label: Unclear*

*Presence: Yes*

*Period: Current*

*- Example 4. (No indication of parole or probation)*

*Text: Veteran does not report any issue with her recent change of meds.*

*Label: Unclear*

*Presence: Unclear*

*Period: Unclear*

*Rule 2 - When 'Period' is 'Future', 'Presence' should be determined carefully. For instance, mention of an upcoming parole hearing does not guarantee parole and hence, 'Label'='Parole', 'Presence'='Unclear', 'Period'='Future'. However, for clear indications such as 'will be granted parole in two weeks', 'Label'='Parole', 'Presence'='Yes', 'Period'='Future'.*

*Rule 3 - For negative examples i.e., examples with 'Presence'='No', the 'Label' can be either 'Parole', 'Probation' or 'Unclear', depending on 'Text'.*

*Rule 3.1 - Examples with negative indications of parole or probation, e.g., 'no parole', 'pt was denied probation' etc.*

*Rule 3.2 - Examples with non-legal probation, e.g., employment probation, school disciplinary probation, sports team probation, etc. ('Label'='Probation')*

*Rule 3.3 - Examples with parole/probation related to the patient's family, relatives, or friends, but not the patient themselves. ('Label'='Parole'/'Probation')*

*Rule 4 - Word-based rules:*

*- The use of "release", while more likely related to parole, could also be used in the split sentencing. Without clear indication, 'Label' should be 'Unclear'.*

*- The use of "reintegration" should be assigned to parole if it is clear from the context that the reintegration is an aftermath of legal action.*

*Rule 5 - When there are two correct combinations for ('Presence','Period') - ('Current','No') and ('History','Yes') prioritize ('History','Yes') as it adds more information.*

*Step-by-step instructions:*

*Step 1 - Analyze all five fields of the example very carefully.*

*Step 2 - Based on the definitions and accepted values for 'Label', 'Presence' and 'Period', evaluate their correctness. Use a 4-point likert scale rating for your evaluation:*

*Rating 1: 'Presence' is incorrect.*

*Rating 2: 'Presence' is correct, but 'Label' and 'Period' are incorrect.*

*Rating 3: 'Label' and 'Presence' are correct, but 'Period' is incorrect.*

*Rating 4: All three fields - 'Label', 'Presence' and 'Period' are correct.*

*Step 3 - Provide a reason explaining your evaluation.*

*Step 4 - Provide the correct values for 'Label', 'Presence' and 'Period'.*

*Example to evaluate:*

*{example}*

*Now think step-by-step. Provide your evaluation in JSON format, as follows:*

*{*

*'Rating': 'An integer on a scale of 4',*

*'Reasoning': 'Your reasoning for the rating provided above',*

*'Label': Corrected 'Label',*

*'Presence': Corrected 'Presence',*

*'Period': Corrected 'Period'*

*}*

## 4. Prompt for Llama 3.3 70B Instruct

Here we only provide the prompt for the best setting - COT + Few-shot. Prompts used for other unsupervised settings are available in the provided code-repository.

*You are tasked with labeling a clinical text snippet as parole, probation or unclear.*

*Definition of Parole: Parole entails the conditional release of a prisoner before completing their maximum sentence, granted by a parole board or similar authority. This release is contingent on demonstrated rehabilitation and the ability to reintegrate into society without posing a threat. The primary objective is to provide supervision and support, aiding offenders in a successful transition back into the community. Parolees serve their time in the community and can be returned to prison for violating any of the conditions of parole imposed by the Board of Parole Commissioners.*

*Definition of Probation: Probation is an alternative to incarceration that allows convicted individuals to stay in the community under probation officers’ supervision. Unlike parole, probation is often part of the original sentence and is not granted after serving time in prison. It serves purposes such as punishment, rehabilitation, and community protection, giving offenders a chance to address their behavior while remaining part of the community. Probation is very similar to parole except that probationers have not served prison time on their suspended sentence and the sentencing judge maintains jurisdiction over the probationer.*

*Given the text (a clinical note or text snippet form it), you should provide the followings:*

*- Label: 'Parole' for mention of parole, 'Probation' for mention of legal probation, or 'Unclear' for ambiguous examples.*

*- Presence: 'Yes' if text is indicative of parole or legal probation, 'No' if not, or 'Unclear' if can not be determined.*

*- Period: 'Current' for ongoing situations, 'History' for past events, 'Future' for future events, 'Unclear' for uncertain periods.*

*Sample examples:*

*{examples}*

*Guidelines:*

*Rule 1 - Set 'Label' to 'Unclear' for unclear or ambiguous situations. Examples:*

*- Example 1.*

*Text: The review of the patient's file indicates no history of parole or probation.*

*Label: Unclear*

*Presence: No*

*Period: History*

*- Example 2.*

*Text: The patient recalls wearing an electronic monitoring device for six months ten years ago, as required by his then-release terms.*

*Label: Unclear*

*Presence: Yes*

*Period: History*

*- Example 3.*

*Text: Veteran has been under strict supervision for two months.*

*Label: Unclear*

*Presence: Yes*

*Period: Current*

*Rule 2 - A text can have multiple presence-period combinations. When both 'Presence'='Yes' and 'Presence'='No' are correct depending on the 'Period' (ex. No/Current and Yes/History are both correct), prioritize 'Presence'='Yes' over 'Presence'='No' as it adds more information and choose the 'Period' appropriately. In particular, priority of 'Presence': Yes > Unclear > No.*

*Rule 3 - When 'Period' is 'Future', determine 'Presence' carefully. For instance, mention of an upcoming parole hearing does not guarantee parole and hence, 'Label'='Parole', 'Presence'='Unclear', 'Period'='Future'. However, for clear indications such as 'will be granted parole in two weeks', 'Label'='Parole', 'Presence'='Yes', 'Period'='Future'.*

*Now label the following clinical text enclosed by <text> </text> tags.*

*<text>*

*{note}*

*</text>*

*Let's think step-by-step. Provide your answer in the following format:*

*<thinking> (your step-by-step thought process) </thinking>*

*<label> (Parole / Probation / Unclear) </label>*

*<presence> (Yes / No / Unclear) </presence>*

*<period> (Current / History / Future / Unclear) </period>*

*<reasoning> (brief rationale for the label, presence, and period values chosen above) </reasoning>*

*Only provide your formatted answer.*

## 6. Annotation Guidelines

1. Assign the period based on when the parole/probation-related event or status applies. Use the following categories:

- *Current*: Use this for events or statuses occurring at present or in the recent past that are still relevant to the current situation.
- *History*: Use this for events that occurred in the past and are no longer affecting the current situation. Treat past events as History without applying a specific one-year cutoff.
- *Future*: Use this for events or statuses that are expected, planned, or may occur in the future.
- *Unclear*: Use this when you cannot determine the period from the available context.

2. Assign the parole/probation label based on the available context. Use *Parole* when the text clearly refers to parole and *Probation* when it clearly refers to probation. If you cannot reliably distinguish between the two, use *Unclear* rather than making an unsupported distinction. For example:

- "He is currently meeting with his parole officer." → Parole
- "She is required to report to her probation officer." → Probation
- "He has restrictions related to his court supervision." → Unclear

3. Do not assume that every occurrence of "parole" or "probation" represents the patient's parole or probation status. Use the surrounding context to determine whether the reference concerns the patient and whether it represents legal supervision. For example:

- "He was placed on academic probation last semester." → Do not interpret this as criminal justice probation.
- "She is in a probationary period at her new job." → Do not interpret this as criminal justice probation.

4. When a parole/probation mention refers to someone other than the patient, indicate that via the *Not_patient* binary indicator. Do this regardless of whether the *Presence* value is Yes, No, or Unclear. For example:

- "He mentioned his neighbor's ongoing struggles with meeting probation requirements." → *Not_patient* = 1
- "She shared concerns about her cousin who struggles with adapting to life after parole." → *Not_patient* = 1

5. Use *Presence* = Yes when the text indicates that the patient has or had parole/probation. Use *Presence* = No when the text explicitly indicates that the patient does not have or did not have parole/probation. Use *Presence* = Unclear when the text contains a relevant reference but does not provide enough information to determine whether parole/probation applies to the patient. For example:

- "He is currently on parole." → Yes
- "She has never been on probation." → No
- "The patient discussed his upcoming meeting with the parole board." → Unclear if the text does not establish whether parole will actually be granted.

6. Identify negated mentions as *Presence* = No when the negation clearly applies to the patient. Do not classify a mention as Yes merely because the words "parole" or "probation" appear. For example:

- "He was denied probation." → Presence = No
- "The patient fears going back to jail, although he has never been on parole." → Presence = No

7. Use *Unclear* when the context does not allow you to reliably distinguish parole from probation. References to courts, sentencing, legal restrictions, community supervision, or similar concepts do not automatically indicate either parole or probation. For example:

- "He is under community supervision following his sentence." → Unclear, unless additional context identifies parole or probation.
- "The patient has restrictions related to his sentence." → Unclear

8. Label the word "release" as *Unclear* unless the surrounding context clearly establishes that it refers to parole. "Release" can be used in contexts other than parole, including split sentencing.
9. Assign "reintegration" to Parole only when the context clearly indicates that the reintegration resulted from a legal action associated with parole. For example:

- "He is working on reintegration following his release under parole supervision." → Parole

10. When both Yes and No could be correct because they correspond to different periods, prioritize Yes. For example:

- "The strict curfew sometimes made her feel like she was back on probation."

→ Label: Probation

→ Presence: Yes

→ Period: History

Although No-Current could also be interpreted from the statement, use Yes-History because it is the more informative interpretation.

11. When *Presence* is ambiguous, prioritize the more informative value according to the following order: Yes > Unclear > No. Use this priority when more than one interpretation is reasonable. For example:

- "She has been free from any community supervision for the past decade, maintaining steady employment."

→ Presence: Unclear

→ Period: History

Although No-Current could be interpreted from the statement, use Unclear-History according to the priority rule.

12. When both *Presence* and *Period* are ambiguous, apply the following priority order: Yes-History > Unclear-History > Unclear-Unclear > No-Current > No-History. For example:

- "During the session, he compared the structured environment of the clinic to a probation office."

→ Label: Probation

→ Presence: Unclear

→ Period: Unclear

Although No-Current could be interpreted, use Unclear-Unclear because it is prioritized over No-Current.

13. When *Presence* = Yes and both Current and History could apply, prioritize Current over History. For example:

- "He is on parole right now and this is not his first time."

→ Label: Parole

→ Presence: Yes

→ Period: Current

Although the statement also indicates a history of parole, use Current because the patient is currently on parole.

14. When *Presence* = Unclear and the reference could correspond to different temporal periods, prioritize Current over History or Future. If you cannot determine the period for an Unclear mention, use Current.
15. When *Presence* = No and both Current and History could apply, prioritize Current over History, and History over Unclear. For example:

- "The patient fears going back to jail, although he has never been on parole."

→ Label: Parole

→ Presence: No

→ Period: Current

Although the statement refers to the patient's history, use No-Current according to the priority rule.

16. For future or hypothetical references, distinguish a potential future event from a confirmed parole/probation status. When the text describes a future possibility but does not establish whether parole/probation will actually occur, use *Presence* = Unclear and *Period* = Future. For example:

- "She mentioned applying for parole but did not discuss whether it was granted."

→ Label: Parole

→ Presence: Unclear

→ Period: Future

Similarly, a statement about meeting with a parole board to discuss potential early release should be labeled Unclear-Future when the eventual parole status is not established.

17. When a statement contains both positive and negative interpretations that differ only because of the period, use the combination with the higher priority rather than assigning both. Follow the Presence and Period priority rules above to select a single annotation.
18. Record the quality according to the following rating criteria provided for the generated examples.

- 1 = *Presence* is incorrect
- 2 = *Presence* is correct, but other things are incorrect (*Reasoning*, *Period*, *Label*)
- 3 = Everything is correct except the Label changing to "unclear" OR GPT's *reasoning* needs a bit of work (but is generally correct)
- 4 = Everything is correct the way GPT assigned it

19. When making an annotation, use the full available context rather than relying on individual keywords. In particular, consider whether the mention is:

- referring to the patient or another person;
- affirmative, negated, or uncertain;
- related to criminal justice supervision rather than a non-legal use of the term; and
- current, historical, future, or temporally unclear.

20. When you cannot confidently determine the parole/probation status or temporal period from the available context, use Unclear rather than making an unsupported inference. When multiple interpretations remain possible, apply the priority rules above to select the most informative annotation.

## 7. Data Stats

Table 1. Dataset Statistics.

| **Text characteristics** | **Synth-PPD (n=7,579)** | **VA_PPD (Segmented)**  **(n=5,341)** | **SMD** |
| --- | --- | --- | --- |
| Word count, mean (SD) | 15.23 (2.32) | 58.28 (69.33) | -0.878 |
| Sentence count, mean (SD) | 1 (0.01) | 2.98 (0.62) | -4.499 |
| Character count, mean (SD) | 94.23 | 403.99 | -0.763 |
